# An output evaluation of a health research foundation’s enhanced grant review process for new investigators

**DOI:** 10.1186/s12961-017-0220-x

**Published:** 2017-06-19

**Authors:** Gregory W. Hammond, Mê-Linh Lê, Tannis Novotny, Stephanie P. B. Caligiuri, Grant N. Pierce, John Wade

**Affiliations:** 1 Department of Medical Microbiology and Medicine, University of Manitoba, 510 Basic Medical Sciences Building, 745 Bannatyne Avenue, Winnipeg, MB R3E 0J9 Canada; 2 Manitoba Medical Service Foundation, 599 Empress Street, Winnipeg, MB R3G 3P3 Canada; 3 Neil John Maclean Health Sciences Library, University of Manitoba, 727 McDermot Avenue, Winnipeg, MB R3E 3P5 Canada; 4 Department of Physiology and Pathophysiology, University of Manitoba, Winnipeg, Canada; 5 Institute of Cardiovascular Sciences, Albrechtsen Research Centre, St. Boniface Hospital, 351 Taché Avenue, Winnipeg, MB R2H 2A6 Canada; 6 Department of Anesthesia & Perioperative Medicine, University of Manitoba, 364 Montrose Street, Winnipeg, MB R3M 3M8 Canada

**Keywords:** Enhanced grant review process, New investigator research funding, Output evaluation, Research productivity, Bibliometric analysis, Effect of health research funding, Economic return on investment, Manitoba Medical Service Foundation, Granting foundations

## Abstract

**Background:**

We assessed the ability of the Manitoba Medical Service Foundation (MMSF, a small not-for-profit foundation affiliated with Manitoba Blue Cross) to determine the best candidates for selection to receive research funding support among new researchers applying to the Research Operating Grants Programme (ROGP).

**Methods:**

Using bibliometric and grants funding analyses, we retrospectively compared indices of academic outputs from five cohorts of MMSF-funded and not MMSF-funded applicants to the annual MMSF ROGP over 2008 to 2012, from 1 to 5 years after having received evaluation decisions from the MMSF enhanced grant review process.

**Results:**

Those researchers funded by the MMSF competition (MMSF-funded) had a statistically significant greater number of publications, a higher *h*-index and greater national Tri-Council (TC) funding, versus those not selected for funding (not MMSF-funded). MMSF-funded applicants and the Manitoba research community have created a strong and rapid (within 1 to 5 years of receiving the MMSF grant) local economic return on investment associated with the MMSF ROGP that supports new investigators, of approximately nine-fold for TC grants by the principal investigator, and of 34-fold for the principal investigator on collaborative (total) TC grants.

**Conclusions:**

The use of small amounts of seed money for competitive research grants at early stages of an MMSF-funded applicant’s career correlates with future short-term success of that applicant. The ability to correctly select promising candidates who subsequently demonstrate greater academic performance after the MMSF funding shows the selection process and the ROGP to be of merit. Multiple components may have contributed to this outcome, including a direct presentation and interview process of the candidate with five-person selection subcommittees, plus an assessment by an external reviewer (the enhanced grant review process). The selection methods used here may add value to the research grant selection processes of new researchers.

## Background

Not-for-profit foundations that fund research grants wish to create value for society [1]. The selection of the best grantees in grant competitions is a key component of the added value created by foundations, but few foundations evaluate their results in a systematic way [1].

The Manitoba Medical Service Foundation (MMSF) was created in 1971 as a not-for-profit foundation affiliated with the Manitoba Blue Cross to support and fund health research and education in Manitoba, Canada, and has contributed over C$20,608,000 to date (May 2017). It has funded health research operating grants since 1974, through annual competitions in the MMSF Research Operating Grants Programme (ROGP). Its primary focus is to fund new researchers, within the first 3 years of their initial academic appointment. A special feature of the ROGP review process is an interactive face-to-face presentation by the applicant with a selection panel subcommittee, in addition to the review of the applicant’s written proposal and an external written review, considered by the same subcommittee. Our experience is that the direct presentation by the candidate, together with an associated question period by a five-person review panel (‘enhanced grant review process’) improves each subcommittee’s understanding of the research proposal, enables a more complete assessment of the candidate and their ideas and communication skills, and therefore is an important addition to the overall selection and decision-making process by the panel.

It has been noted in a recent review on grant panel decision-making [2] that there is “*hardly any research on predictive validity of panel decisions, especially early career applicants – are they the best researchers on looking back after 10 years?*”. MMSF wished to evaluate the outputs of recent research grant applicants selected by the grant review subcommittees to assess their decisions over a multi-year period. Our hypothesis was that the MMSF grants review and adjudication process selects the best candidates among new applicants to the ROGP. Therefore, MMSF sought to determine research productivity by objective measures, consistent with guidelines of the Canadian Academy of Health Sciences [3]. We used bibliometric and research funding analysis to determine whether the MMSF processes successfully selected the best candidates through the annual MMSF ROGP review competitions, over five recent competitions for which national granting agencies’ research bibliometric and funding data were available electronically. The bibliometric indicators aimed to assess ‘advancing knowledge’ through two of the four subcategories, namely a quality indicator and an activity indicator [3]. Research funding analysis was used as a capacity-building indicator [3], and also served as an additional objective measure used to assess outputs of the candidates in the competitions.

The study aims were to answer two questions:

1. Does the MMSF grant review and decision-making process select those candidates who subsequently demonstrate a greater research productivity versus those who were not MMSF-funded?
2. What was the economic return on investment effect (ROI) after MMSF funding, as determined by evidence of funding received into the Manitoba research community by MMSF grant applicants, based upon their funding success from Tri-Council (Canadian Institutes of Health Research (CIHR), Social Sciences and Humanities Research Council (SSHRC), and Natural Sciences and Engineering Research Council of Canada (NSERC)) Canadian granting organisations?

## Methods

### Setting: MMSF grant review processes

The MMSF ROGP process and evaluation framework are posted on the MMSF website [4]. Approximately 20 to 35 grant applications are received annually. Applications are welcomed from medical and scientific researchers and allied health professionals involved in preventive, therapeutic or rehabilitative care, who promote scientific and educational activities in the maintenance and improvement of the health and well-being of the people of the Province of Manitoba.

All applicants are from postsecondary institutions, almost all from the University of Manitoba. During this study, the applicants were within 3 years of establishing themselves as independent researchers in Manitoba. An independent researcher is autonomous regarding their research activities and has a Manitoba academic or research appointment that allows the individual to pursue the proposed research project, to engage in independent research activities for the entire duration of the funding, to supervise trainees, and to publish the research results. The MMSF defines the start of the 3 years of grant eligibility to begin when the applicant received their first academic appointment in any province or country. The researchers who were eligible for the MMSF ROGP competition were those who were recruited by their academic institution, from a range of faculties. We do not have the complete academic dossier on the background training of each of the candidates, as this resides within each faculty. We do not capture the age of the candidates. Eligible researchers may include residents and fellows (residents and fellows are not required to have an academic or research appointment). There were 10 residents and fellows who applied over the study period.

The MMSF Board is composed of approximately 30 volunteer members, with approximately one-third being community representatives and two-thirds being academic health leaders [4]. An Awards Assessment Committee oversees the education and grants awards process. The annual operating grants competition involves the selection of subcommittees for each of the eligible applicants. Each subcommittee is composed of five individuals from the Board, one of whom is usually a community member. Two individuals on each sub-committee are academic leaders, chosen as closely as possible to have expertise in the field relevant to the topic of the grant. The Chair of the subcommittee is chosen by the Executive Director and Administrative Assistant from amongst those three subcommittee members. The other two subcommittee panellists are the Executive Director and the Assistant Executive Director. Both sit on all panels as a resource to optimise overall consistency of the grant reviews. Each interview consists of a 15 minute presentation by the candidate, then a question period. Requested budgets are also reviewed and justification is sought for selected items. The candidate is then excused while the Subcommittee discusses the grant and adjudicates the score of both the written grant and the presentation, according to the criteria that are outlined in the Application form. The adjudication includes reviewing the assessment of an external evaluator, who is selected from a list of individuals from the MMSF database of external reviewers, or from the names provided on the submitted applications. The panel grades the excellence of the proposal, on the Project Point Scale, to determine the score from 1 to 20 points, with 16 to 20 being excellent, 11 to 15 being very good, 6 to 10 being good, and 5 or less being poor. The MMSF subcommittee members for each candidate agree by consensus about the ratings to be awarded for each candidate at the conclusion of each assessment. Although each subcommittee chair and two other members of each subcommittee are different for each candidate’s review, some consistency is provided by the inclusion of the Executive Director and the Assistant Executive Director in all of the subcommittee assessments. After the presentation, the candidate is given direct feedback on their application and presentation, with the aim of helping to advance the candidate’s career experience and to help strengthen the proposal, but no commitment to funding is made at that time. Scores are assigned after each presentation and overall adjudication rankings are determined by the Executive Director and Assistant Executive Director after all of the applications have been reviewed. Budgeted funding amounts and the quality of the proposal determine the initial cut-off for MMSF-funded applications, based upon the total points. The overall Operating Grant budget, the applicants’ budget requests, and the number and quality of grants vary each year, so the amounts of each grant vary by year. This usually allows funding of ‘excellent’ and most ‘very good’ quality grants annually. These ranking results are defined on our Applications for Operating Grant Application Forms, which include scores of 11 points or more out of 20 for Project Point Scale scores. The Awards Assessment Committee then reviews the overall list and makes recommendations to the MMSF Board for final approval. Candidates are notified by the end of the calendar year about the outcome of each grant application, with MMSF-funded grants starting on April 1 of the following year. MMSF research grants awarded are not greater than C$35,000 and are for a 1-year period only.

### Design and methodology for assessment of outputs

To test the hypothesis that the MMSF selection process chooses the best applicants, academic outputs of all new researcher applicants (defined as within the first 3 years of their first academic appointment) were analysed comparing MMSF-funded to not MMSF-funded candidates. In order to determine a researcher’s subsequent academic outputs after being MMSF-funded, two different areas were examined, namely publication history and funding history. Publication history involved determination of the number of journal articles published, as well as the author’s *h*-index. An *h*-index is a numeric measurement that captures information related to the productivity and impact of a researcher using the number of papers an author has published, as well as the number of subsequent articles that cite the author [5, 6]. While there are criticisms of the *h*-index [7, 8], it is highly used by researchers, academic institutions and funding agencies as a valid and common measure of scientific publishing impact of researchers. In order to determine funding history, the databases of the Tri-Council granting agencies were searched to determine whether candidates had received subsequent funding and whether they acted as Principal Investigator (PI) or a collaborator. The Tri-Council agencies are the CIHR, NSERC and SSHRC. As the Government of Canada’s health research investment agency, the CIHR supports excellence across all four pillars of health research, namely biomedical, clinical, health systems services and population health. CIHR invests approximately C$1 billion each year to support health research [9]. The NSERC is the largest funder of science and engineering research in Canada. With funding from the Government of Canada, NSERC supports the world-class research of over 41,000 talented students and professors at universities and colleges across the country with an annual budget of C$1.1 billion [10]. The SSHRC is the federal research funding agency that promotes and supports postsecondary-based research and research training in the humanities and social sciences. SSHRC’s budget is determined each year by Parliament. SSHRC’s grants and scholarships budget for 2015–2016 was C$353.3 million [11].

The names of MMSF-funded (n = 88) and not MMSF-funded (n = 30) MMSF grant applicants from five grant competitions over 2008–2012 were compiled into a spreadsheet, and then the above indicators were searched for each applicant, to June 2014, i.e. from approximately 5 years through 1 year post receipt of grant funding for MMSF applicants.

### Publications

In order to make the search manageable, only one database was used to retrieve publication information related to each researcher. Scopus is the largest database of science, technology and medicine research, indexing over 22,000 journals [12]. In addition, Scopus has made a significant effort to collect all of a researcher’s publications under a single name and unique ID number. Data were collected in the spring and summer of 2014 using the function ‘Author Search’ for each researcher name. If located, the associated Scopus Author ID (a unique 10 digit numeric code), the researcher’s *h*-index and the number of publications indexed in Scopus were noted. In very few cases, a researcher had multiple Scopus Author IDs, and therefore multiple *h*-indices. In these cases, the number of publications were added together and a note was made. The Scopus Author ID with the higher *h*-index was used but no attempts were made to determine if the combined publications would result in a changed *h*-index.

### Funding

In order to determine a researcher’s relative success related to funding, a search was done of three different funding agencies and their funded applicants. Details on the databases are in Table 1.

**Table 1:** Funding databases analysed

	CIHR	NSERC	SSHRC
Full Name	Canadian Institutes of Health Research (Tri-Council)	Natural Sciences and Engineering Research Council of Canada (Tri-Council)	Social Sciences and Humanities Research Council (Tri-Council)
Website	http://webapps.cihr-irsc.gc.ca/funding/Search?p_language=E&p_version=CIHR	http://www.nserc-crsng.gc.ca/ase-oro/index_eng.asp	http://www.outil.ost.uqam.ca/CRSH/RechProj.aspx?vLangue=Anglais
Geographic focus	Canadian-based researchers	Canadian-based researchers	Canadian-based researchers
Coverage	2009/10 - 2013/14	1981-2014	1998-2012
Searched by:	‘Investigator’	‘Name of Person’	‘Applicant’
Limitations used	Grants where investigator only acted as a supervisor were eliminated	None	None
Notes		Searched by ‘Competition Year’	Searched by ‘Competition Year’

## Statistics

Individuals with more than one application to MMSF competitions were limited to their MMSF-funded application data point, as otherwise this would violate the assumption of independent values.

All data were tested for normality and homogeneity of variance using the Shapiro Wilk’s test and Levene test, respectively. Two-group comparisons were tested with the Wilcoxon-Rank Sum test due to the non-Gaussian distribution of the publication, *h*-index and funding data. Discrete and continuous data were displayed as mean ± standard error. All tests were set at a significance level of 0.05.

## Results

There were 35 females in the MMSF-funded group of 49 female applicants (71.43% of female applicants were MMSF-funded) versus 53 males in the MMSF-funded group of 69 male applicants (76.81% of male applicants were MMSF-funded). This shows similar selection rates irrespective of the sex of the applicant.

**Fig. 1:**
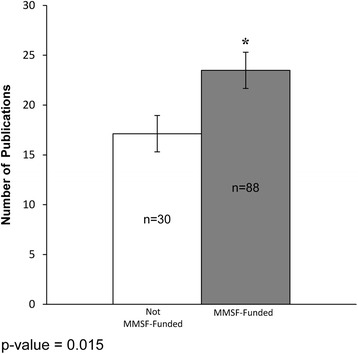
Publications – comparison of research productivity outputs of MMSF applicants

Indices of academic outputs after the MMSF competition were compared by MMSF application success. In a 5 year comparison of the not MMSF-funded versus MMSF-funded applicants, there was a statistically significant difference in the average and median number of publications in the not MMSF-funded (17.13 and 10.00) versus MMSF-funded applicants (23.49 and 20.5) (Fig. 1).

**Fig. 2:**
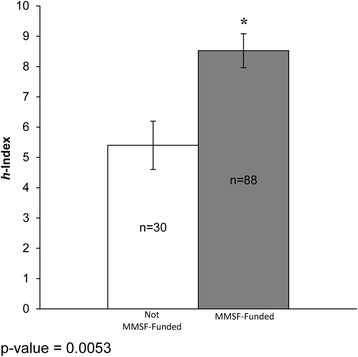
*h*-index – comparison of research productivity outputs of MMSF-funded versus not MMSF-funded applicants

Similarly, there was a statistically significant difference in the *h*-index average and median of the not MMSF-funded (5.40 and 4.5) versus MMSF-funded applicants (8.52 and 7.50), respectively (Fig. 2).

**Fig. 3:**
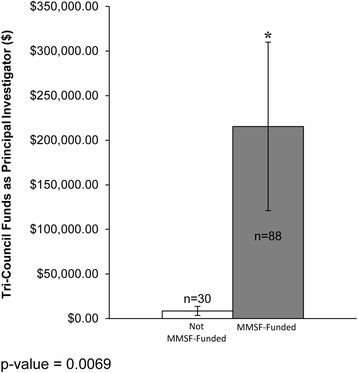
Tri-Council funding as a principal investigator – comparison of research productivity of MMSF applicants as a function of MMSF funding status

**Fig. 4:**
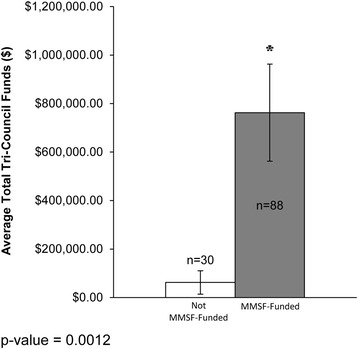
Total Tri-Council funding – comparison of research productivity of MMSF-funded and not MMSF-funded applicants

Regarding external funding obtained through national Tri-Council granting agencies, the average funding of all not MMSF-funded PI applicants was C$8476, versus C$215,432 as the average of all MMSF-funded applicants over the follow-up period to August 2014. Specifically, MMSF-funded PI applicants received on average 12 times more future Tri-council funds versus not MMSF-funded PI applicants (Fig. 3). In Fig. 4, all Tri-Council funding was totalled in these calculations, whether the MMSF applicant was a PI or a Collaborator on the Tri-Council grant (Fig. 4).

**Fig. 5:**
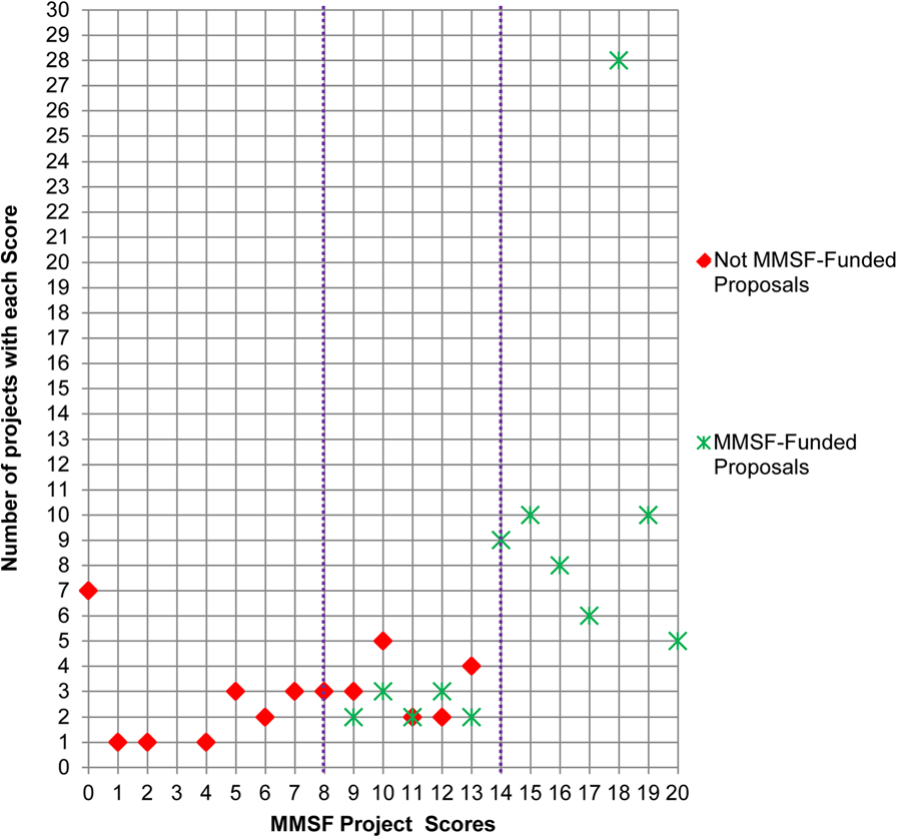
Relationship between project scores and number of projects with each score 2008–2012 of MMSF-funded and not MMSF-funded applicants

The relationship between each project score, its frequency and MMSF funding success is shown in Fig. 5. The graphic distribution of results shows three components separated by dashed lines. Grants with a score of 8 points or less were never funded by MMSF. In the intermediate range of 9 to 13 points, 12 of 28 grants were funded by MMSF, which depended on the available funds and the quality of competition within each year. All grants of 14 points or more were funded by MMSF, which fall into the high ‘Very Good’ and ‘Excellent’ part of the scale.

**Fig. 6:**
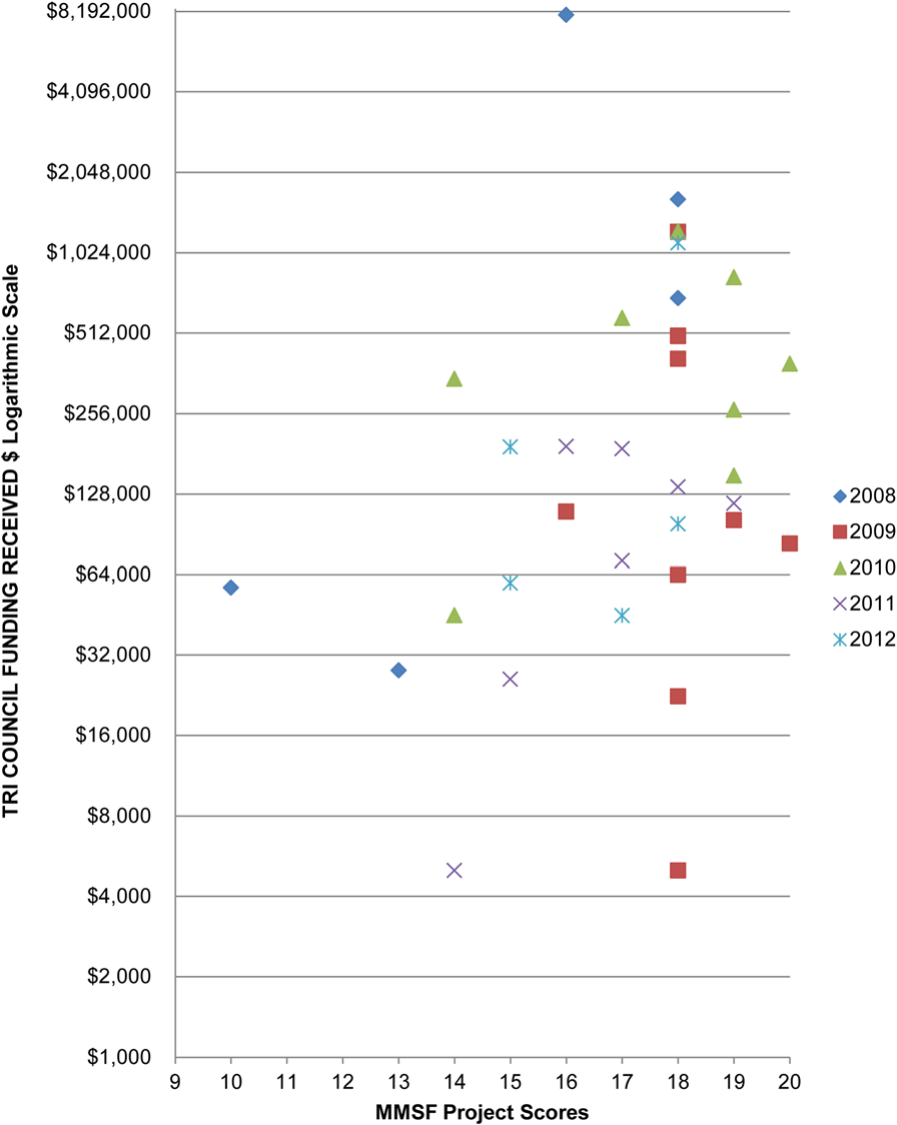
Relationship between MMSF grant project scores and subsequent Tri-Council funding as a Principal Investigator

MMSF-funded Investigators with projects scores below 14 points rarely went on to obtain Tri-Council funding support as a PI (only 2 of 12 applicants), whereas 32 of 76 individuals with scores of 14 or above obtained such funding (Fig. 6).

### Economic ROI

The economic return to the Province of Manitoba from national grant funding received by researchers following MMSF investment in operating grant programme research is summarised for all 5 years studied (Table 2). The total MMSF funding provided for new investigator operating grants to all MMSF-funded applicants over the 2008–2012 5-year competition period was C$1,912,300. In comparison, the Tri-Council funding received as a PI was C$18,958,002 up to June 2014, so the ROI (over this period calculated as the gain from investment minus the cost of the investment divided by the cost of the investment) for all MMSF-funded applicants as PIs on Tri-Council grants was C$18,958,002 minus C$1,912,300 divided by C$1,912,300 = 8.91. The total Tri-Council funding received with the MMSF-fundedapplicant as a Co-Applicant on Tri-Council grants was C$67,099,929, so the ROI for all MMSF-funded applicants in collaborative Tri-council grants was C$67,099,929 minus C$1,912,300 divided by C$1,912,300 = 34.09 over this period.

**Table 2:** Relationship between amount of MMSF funding and Principal Investigator (PI) Tri-Council and total Tri-Council funding

Award Year 2008–2012	Not MMSF-funded		MMSF-funded				
	PI Tri-Council funding received	Total Tri-Council funding received	Total MMSF funding received	PI Tri-Council funding received	ROI	Total Tri-Council funding received	ROI
Tri-Council grants	*n* = 4	*n* = 9		*n* = 34		*n* = 49	
Total	C$254,272	C$1,869,998	C$1,912,300	C$18,958,002	8.91	C$67,099,929	34.09
Average	C$63,568	C$207,778	C$21,731	C$557,588		C$1,369,386	
Median	C$59,250	C$24,973	C$20,000	C$143,000		C$220,150	
Std. Dev.	C$52,764	C$468,090	C$4,104	C$1,369,337		C$2,351,257	

## Discussion

This study sought to answer two questions intended to evaluate the ROGP of competitive start-up operating grant funding by the Manitoba Medical Service Foundation to new health researchers in Manitoba, Canada, from the 2008 through 2012 competitions.

We assessed the outputs of the MMSF ROGP to determine if the best candidates for funding had been selected by investigating indices of future success, i.e. number of publications, *h*-index and Tri-Council funding. Those who were MMSF-funded in the competitions were flourishing more in their career versus the not MMSF-funded candidates. This is evidenced by the greater number of publications, a higher *h*-index and greater amounts of Tri-Council grant funding as a Collaborator and as a PI for the MMSF-funded, as opposed to the not MMSF-funded applicants. It can be concluded that the selection process by the MMSF for the best candidates had merit.

Finally, we have demonstrated that the individuals who have been MMSF-funded through the MMSF operating grants competitions have also been very successful in bringing external Tri-Council funding to the Province of Manitoba through Canadian competitions within 1 to 5 years of their receiving an MMSF operating grant. For the entire operating grant programme over this same period, where the total MMSF grant funding was C$1,912,300, the PIs were funded C$18,958,002 through Tri-Council grants, for a ROI of approximately 9. This is probably an underestimate of the overall potential impact of the MMSF funding, as Tri-Council grants were the only external granting agencies studied and the follow-up period was relatively short. The ROI is further increased to 34 if we compared MMSF funding amounts with the total Tri-Council grant programme results of C$67,099,929. However, we recognise that, although funding by MMSF may have contributed to any later success, we do not attribute later success only to MMSF. Many other factors likely played an important role in the development of these new researchers. In summary, investments in health research through operating grants provided by MMSF to new health researchers in Manitoba have shown a rapid and strong local economic ROI effect within 1–5 years after MMSF funding.

The selection process of the MMSF-funded operating grant recipients is intended to be fair and robust and to contribute to the selection of highly worthy projects and candidates. In addition to the written grant proposal, the opportunity for a direct presentation of the research to a small critical selection panel enables the subcommittees the opportunity to better understand the proposal and the applicant’s ideas. The understanding is also increased by a written external review for each proposal, especially in complex proposals. As the grant review subcommittees are composed of individuals who have a diversity and depth of experience, both lay and professional, the opportunity to interview the applicant directly enables a more complete assessment of the applicant, as well as the project. Ultimately, it is the attributes of the individual applicant that determine their overall success. This direct assessment method, which includes the applicant, is more comprehensive than only a review of a written proposal.

MMSF funded a relatively high percentage (88/125 or 70%) of ROGP applicants. While the funding success rate of the applicants to the MMSF ROGP is high in comparison to the rates of funded applicants to national competitions, the selection of a high percentage of candidates for support by MMSF helps to maximise the number of potential candidates applying for national competitions. Although the total number of applications to Tri-Council granting agencies by the MMSF applicants over this study period is not known, 34 of 88 (38.6%) of the MMSF-funded individuals were funded as PI applicants to Tri-Council competitions within 1–5 years of their MMSF grant, versus only 4 of 30 (13.3%) of those not funded by MMSF. In comparison, the annual successful CIHR grant competition funding rate was 23% in 2010–2011 [13]. The distribution of seed funding more widely among new investigators may have encouraged their research careers, which may have contributed to additional national grant funding for these new investigators.

The success of MMSF grantees as PIs in achieving national funding support is likely due to many factors beyond the receipt of the MMSF grant funding. There is very strong and co-operative support for new investigators in our relatively small academic community, including by universities, colleagues, collaborators and mentors, so that, once selected and given ‘seed funds’ such as those of the MMSF, the PIs can be successful. The value of investing in the early research training of both medical and dental students has been previously demonstrated at the University of Manitoba by results of academic career outputs, through participation in the BSc (Med) and the BSc (Dental) programmes [14, 15]. In summary, MMSF-funded applicants and the Manitoba research community have created a strong local economic ROI effect, as well as the benefits of the research for both health and personal development.

Are there potential lessons that can be learned or may be validated from the experience of the MMSF that may have applications for larger granting organisations? The MMSF has utilised five-person review subcommittees for over 40 years. Recently, the CIHR conducted an analysis of post-doctoral fellowship competitions and determined that five persons was the optimal size for grant review committees [16]. In regards to grant review processes that involve direct candidate interviews, the Canadian Networks of Centres of Excellence 2013 Knowledge Mobilization Initiative grants competition required, for the first time in Stage III of full applications, that the Network Director or applicant would be present for a face-to-face meeting with the Networks of Centres of Excellence Standing Selection Committee [17]. Both the Danish Research Council and the European Research Commission utilise a direct interview process, bringing candidates for interviews as part of a two-stage evaluation process [18, 19]. Our data support similar practices. Overall, additional research is required on the most effective and efficient judgement and decision-making processes in groups, as it applies to panel peer review of grant applications [20].

There were a number of limitations with this project. Firstly, it is possible that some publications or grants may have been written or awarded prior to the researcher receiving an MMSF award. It was not possible to separate out publications prior to the grant, as this would have made obtaining an *h*-index impossible. In addition, the often protracted process of completing a research project also means a project could have been conceived of, completed, and written up prior to receiving a grant, but only published afterwards. However, as MMSF ROGP researchers were at the early stages of their career, this was not deemed to be a major issue, as most of these researchers would have had a minimal number of prior publications and would have been unlikely to have received any previous national grants, particularly as a PI.

As noted above, when the Author Search in Scopus was completed, there were occasional instances where a researcher did not have a unified profile with a single *h*-index and combined publications. Instead, their publications were spread out over two or more Scopus Author IDs. In these instances, the number of publications were combined and the higher *h*-index was noted, but no attempts were made to determine if the combined publications would affect the author’s *h*-index. It is important to note that these cases were rare and in all instances would only result in a researcher having a higher *h*-index, not lower.

The lack of funding from an MMSF grant early in a research career may itself inhibit research progress and could therefore be a negative influence on a subsequent academic career. We have not examined whether the individuals studied here received other grant awards that may have contributed to their productivity either before or after receiving an MMSF operating grant. The number of MMSF candidates was relatively small, so large funding awards from national granting agencies to a few individuals can increase the average amount received by each individual. While a maximum of just over 5 years of follow-up in this study post funding is not a long period of assessment, it was limited by the availability of CIHR electronic data which began in 2010, so the grant recipients had only 1–5 years of follow-up after receipt of funding for the assessments conducted in this study. The overall funding results for individuals followed in this study is likely an underestimate of the total funding received, as we have not included funding from foundations and granting organisations outside of the Tri-Council national granting agencies of the federal government and the follow-up period was relatively short. None of the applicants received funding from the National Institutes of Health, but no other international funding organisations were studied.

Finally, it is possible that some publications and funding amounts of total grant funds received may be captured twice in the analysis, as it is possible that some of the MMSF researchers may have collaborated together across different years in team grants. It was not possible to search for or remove these possible collaborations on national grants so that they were only captured a single time. However, the PI output analysis would not have any duplication of funding amounts, as that funding is specific to each PI.

## Conclusion

Bibliometric and grant funding analyses have demonstrated that an enhanced grant review process, which includes a presentation and a direct interview of each candidate by five-person subcommittees and an external review, has selected the most productive individuals for grant funding. Small research grants awarded early in the careers of new investigators can help to yield a very strong and rapid local economic ROI effect.

## Abbreviations

CIHR: Canadian Institutes for Health Research
MMSF: Manitoba Medical Service Foundation
NSERC: Natural Sciences and Engineering Research Council
PI: Principal Investigator
ROGP: Research Operating Grants Program
ROI: return on investment
SSHRC: Social Sciences and Humanities Research Council
